# Emotional-based pedagogy and facilitating EFL learners' perceived flow in online education

**DOI:** 10.3389/fpsyg.2022.960287

**Published:** 2022-09-14

**Authors:** Parisa Abdolrezapour, Nasim Ghanbari

**Affiliations:** ^1^Department of English, Salman Farsi University of Kazerun, Kazerun, Iran; ^2^Department of English Language and Literature, Faculty of Humanities, Persian Gulf University, Bushehr, Iran

**Keywords:** emotional intelligence, flow, perception, online education, EFL learners

## Abstract

Given the fundamental role of emotional intelligence (EI) in learning, especially in virtual learning contexts where individuals experience more stress and anxiety, the need to understand and recognize one's own feelings and the mutual feelings of peers has gained more importance. Flow as the ultimate state in harnessing emotions in the service of performance and learning has been introduced as the main reason for one's willingness to perform activities which are connected to no external motivation. In this regard, the present study was conducted to first introduce a new educational program to enhance the EI level in the English as a foreign language (EFL) online education environment and next to investigate the possibility of raising EFL learners' perceived flow. To achieve these goals, the study recruited a sample of 67 EFL learners who were next divided into experimental (*n* = 32) and control (*n* = 35) groups. The experimental group received the EI intervention over 10 weeks and the control group received the ordinary online EFL instruction. Data were collected through EI and flow questionnaires and semi-structured interviews which focused on learners' perception of the EI intervention and signs of enhanced flow. Statistical analysis of the data showed a positive effect of the program on the learners' EI and their perceived flow. The study emphasizes the role of applying positive emotions in making language learners more engaged in online classroom tasks.

## Introduction

Along with the global outbreak of COVID-19 and the following social distancing and the physical gap between people as a strategy to decrease the dissemination of the disease, many businesses shifted to online platforms and schools closed their doors. Accordingly, many institutions and educational settings opted for alternative methods of teaching and learning process to cope with this dramatic change and online instruction delivery gained more attention. Such a change in the routine behavior may have profound effects on the learners' perceptions, attitudes, and feelings as they have to not only accept the situation but also adopt themselves and try to continue their routine lives. The imbalance brought to life and the difference caused in the type of emotions felt made some learners detached from their paths of meaning and purpose and resulted in a lack of focus on tasks and activities presented in online classes. In addition, the current lockdown was associated with slowing down of time, which is thought to be brought by boredom and sadness (Droit-Volet et al., 2020; Li and Dewaele, 2020; Resnik and Dewaele, 2021). As these changes in individuals' affect are hypothesized to have lasting negative impacts (Chadi et al., 2022; Samji et al., 2022), a number of researchers focused on ways to reduce boredom and improve emotional well-being (e.g., Rankin et al., 2019; Patsali et al., 2020).

The literature investigating learners' psychological factors during this lockdown has focused on such factors as learners' mental health (Patsali et al., 2020; Saha et al., 2020; Shigemura et al., 2020), learners' attitude (Unger and Meiran, 2020), emotion regulation (Restubog et al., 2020), boredom (Derakhshan et al., 2021b; Kruk et al., 2022), and anxiety and coping strategies (Hyland et al., 2020; Savitsky et al., 2020). One of the variables related to anxiety and motivation is flow which has been the focus of few number of studies (Dewaele et al., 2022; Wang and Huang, 2022) in educational contexts especially during this pandemic. Flow is a state characterized by the experience in which one is subconsciously immersed in an activity, have active participation in learning tasks with a feeling of pleasure, and nothing but the action would be of importance. So, following previous studies which showed the effectiveness of flow for boosting emotional states during the stressful periods of uncertainty (Rankin et al., 2019), two promising candidates for the successful adaptation to online education could be emotional intelligence and flow. Therefore, applying a mixed-methods design, the present study aimed to investigate the effectiveness of introducing emotional activities to boost the learners' perceived flow.

## Review of literature

### Emotional intelligence

Emotions, and more specifically positive emotions, and their influential role in learning a second language was quite a neglected area of enquiry till the last three decades (Dewaele and Li, 2020; Wang et al., 2021). However, since the 2010s, following the establishment of the International Association for the Psychology of Language Learning, there was a surge of interest in positive and negative emotions in second/foreign language contexts (Abdolrezapour, 2017a,b; Dewaele et al., 2018; Derakhshan et al., 2021a; Ghanbari and Abdolrezapour, 2021) and various definitions have been proposed for emotions consistent with the researchers' viewpoints. In this article, we will use the definition proposed by Reeve (2009) as “short-lived subjective-physiological-functional-expressive phenomena that orchestrate how we react adaptively to the important events in our lives” (p. 9), which attribute a multidimensional, complex nature to emotions as subjective feelings and social phenomena. And the focus is on academic emotions, defined as emotions that are “directly linked to academic learning, classroom instruction, and achievement” (Pekrun et al., 2002, p. 91).

Currently, the two most widely investigated emotions in the language learning context are foreign language enjoyment (FLE) and foreign language classroom anxiety (FLCA) (Resnik and Dewaele, 2020). In this respect, Dewaele and Alfawzan (2018) attention was drawn to the link between FLE and FLCA and language learners' performance and found that FLE was a better predictor of performance than FLCA, which implies that it is crucial for language teachers to put premium on boosting learners' positive emotions to ensure enhanced acquisition. This being the case and considering the relationship found between FLE, FLCA, and emotional intelligence (Li and Xu, 2019; Resnik and Dewaele, 2020), and the malleability of EI (Abdolrezapour, 2017a,b), the best way to help learners regulate their emotions is through the field of emotional intelligence (Goetz and Bieg, 2016).

The most broadly acknowledged and widely accepted definition of emotional intelligence (EI), considered as a facet of general intelligence, has been proposed by Salovey and Mayer in 1990, who defined emotional intelligence as “the ability to monitor one's own and others' feelings and emotions, to discriminate among them, and to use this information to guide one's thinking, and actions” (Salovey and Mayer, 1990, p. 189). This investigation follows the Goleman's EI framework (2005), according to which EI is composed of five characteristics: (1) knowing one's emotion, (2) managing one's emotion (i.e. handling fear, anxiety), (3) motivating oneself (emotional control, the ability to delay gratification), (4) recognizing emotions in others, and (5) handling relationships.

Considering the positive link between emotional intelligence and learners' academic performance and the social nature of emotions (Reeve, 2009; Ghanbari and Abdolrezapour, 2021), there is a general agreement among scholars that nurturing learners' emotional intelligence allows them to be connected with others and learn in a more effective way and increase their chances of success both in school and future life (Goetz and Bieg, 2016). Accordingly, if class activities are emotionally and personally relevant to students' lives, corresponding to the characteristics of emotional intelligence, they would engage learners' attention, encourage cooperative learning and would lead to deeper learning and more commitment to learning processes (Abdolrezapour and Tavakoli, 2012).

As in each subject and context, learners' emotional intelligence was found to be prominent (e.g., Domitrovich et al., 2017; Corcoran et al., 2018), in an online setting, learners' emotion-related personality traits are influentially engaged. So, researchers' attention was attracted to the contributions of learners' and teachers' emotional intelligence to their performance in a language education context (Buzdar et al., 2016; Alenezi, 2020; Li and Dewaele, 2020; Fraschini and Tao, 2021; Zhao and Song, 2022). In this regard, Derakhshan et al. (2021b) confirmed that EFL teachers and students encounter more difficulties in online classes and their teacher participants pointed to the importance of EI in online educational contexts. In the same vein, Buzdar et al. (2016) confirmed the large predictive contribution of students' emotional intelligence in explaining variance in their readiness for online education. Fraschini and Tao (2021), acknowledging the need to investigate EFL learners' emotion in a virtual context, pointed to the link between positive emotions and learning achievements with some teacher characteristics. As for the possibility of enhancing learners' EI in a foreign language learning context, Abdolrezapour (2017b) studied the role of emotions in computer-mediated learning and provided evidence that emotions could be successfully exploited in EFL classrooms through a number of activities presented by the language instructor. Following this line of research, and Zhao and Song (2022), who found differences between learners' emotions of face-to-face classes and those of online learning, the current attempt aimed to shed light on the effectiveness of emotional intelligence in an online education context and its relation to the learners' flow.

### Flow

Flow is described as “a subjective state that people report when they are completely involved in something to the point of forgetting time, fatigue, and everything else but the activity itself” (Csikszentmihalyi and Rathunde, 1993, p. 59). It refers to a state of optimal experience or peak performance in which the individual has an intense engagement in an activity. This state has been linked to the feeling of enjoyment and pleasure and has been introduced as the main reason for one's willingness to execute actions which are linked to no external motivation; the leading cause of which was known to be an internal locus of control, a personality construct that refers to people's belief about the action-outcome relationship (Rotter, 1966). According to Goleman (1995), flow state is emotional intelligence at its best and it is the ultimate possible state in harnessing the emotions in the service of performance and learning. In such status, one would experience positive emotions aligned with the task at hand.

This concept has been found influential in various fields including sports (as mentioned in a review by Swann et al., 2012), gaming (e.g., Bressler and Bodzin, 2016), research activities (Hudock, 2015), and work-related activities (e.g., Nakamura and Csikszentmihalyi, 2009). Khan and Pearce (2015) correlated this state with concentration, intrinsic motivation, and enjoyment to perform actions at hand, which would affect learners' perceived learning (Hung et al., 2015) and satisfaction (Buil et al., 2018). Furthermore, in educational contexts, the state of flow has been regarded as a prerequisite of tasks demanding higher-order thinking, in that it provides a higher level of concentration and focus on the task and various studies pointed to its correlation with learning outcomes (e.g., Everett and Raven, 2015).

Following the pioneering study of Egbert (2003), who pointed that the state of flow involves a particularly intense focus in an activity to the extent that one may even lose self-consciousness and a track of time, a number of researchers focused on the efficiency of flow in the foreign language context (e.g., Aubrey, 2017; Dewaele and MacIntyre, 2019; Liu and Song, 2021; Dewaele et al., 2022; Zhao and Khan, 2022). In her study, Egbert (2003) maintained that some preconditions must exist for the flow experience to occur including (a) a balance of skills and challenge, (b) intense concentration, (c) clear goals, (d) immediate feedback, (e) a sense of control, and (f) interest. Later, studying the experience of positive flow and anti-flow by 232 Spanish foreign language learners from around the world, Dewaele and MacIntyre (2019) pointed to more experience of positive flow, which was linked to a higher degree of multilingualism, high relative standing in the group, age, and years of study.

A series of empirical studies also examined flow in the e-learning contexts (Rodriguez-Ardura and Meseguer-Artola, 2017; Li et al., 2021; Liu and Song, 2021; Wang and Huang, 2022; Zhao and Khan, 2022) which shows the importance of providing learning programs that raise the students' flow states as a key feature for academic success in the virtual education environment. In this regard, Pearce (2005) investigated the possibility of making e-learning activities motivating and engaging, yet still productive. He applied two different methods to measure flow experiences and pointed to the dynamic nature of the students' flow experiences. More recently, Wang and Huang (2022) developed a 14-item foreign language flow scale in a Chinese context and found that the flow state is composed of three dimensions including enjoyment, boredom, and anxiety. However, the online education, especially during the COVID pandemic, was found to be more accompanied by students' boredom and different variables have been found influential in this regard including the topics assigned, activity types (i.e., repetitiveness and monotony of activities used), excessive teacher control, lack of learner participation, as well as over-challenging tasks (Derakhshan et al., 2021b; Zawodniak et al., 2021; Kruk et al., 2022).

In general, previous studies have all pointed to the importance of flow in various learning contexts. It was found that it would lead to learners' interest and satisfaction in the tasks and their desire to experience more challenging tasks. However, applying tasks that enhance learners' flow had been a neglected area of study in foreign language learning situations. Flow or the sense of optimal state is linked to the peak performance and subsequent happiness which points to the higher correlation with affective rather than cognitive aspects of flow-related processes. Thus, following Derakhshan et al. (2021b), who proposed adopting livelier class, more teacher-student interactions, and improved interpersonal relationships to lower students' boredom in EFL online classes, incorporating emotional training programs in this investigation is hypothesized to support enhanced flow.

### Emotional intelligence and flow

Flow state is clearly a key construct of positive psychology and a highly emotional experience. As already noted, the flow experience is characterized by a condition in which the individual is completely immersed in actions with a high degree of intrinsic motivation and it has been found related to some positive experiences including well-being, enjoyment, affect, and satisfaction. It is now well-established from a variety of studies that EI and flow are linked (Culver and Yokomoto, 1999; Li and Dewaele, 2020; Wang and Shaheryar, 2020; Rakei et al., 2022). In this part of the study, we wish to discuss a number of flow-related studies dealing with a wide range of concepts associated with different components of emotional intelligence as proposed by Petrides et al. (2006), including well-being, self-control, emotionality, and sociability.

There is a consensus among psychologists that emotional psychological well-being (Csikszentmihalyi, 2014; Rankin et al., 2019; Lynch and Troy, 2021) is positively linked to one's experience of flow. Moreover, Kuhnle et al. (2012) pointed that self-control is a good predictor of flow experiences in eighth graders. This being the case, Rivkin et al. (2018) found that higher levels of flow experiences would enhance self-control demands and would ultimately result in higher well-being. The authors also claimed that the experience of flow revealed higher levels of intrinsic motivation (Rivkin et al., 2018). Finally, as for social skills, Walker (2010) examined whether the social flow was preferred to solitary flow and the results of his survey study revealed that social flow was more enjoyable than solitary flow. The author concluded that students like to do the tasks together rather than alone. In addition, social flow experiences were found to be stimulated by positive collective gatherings (Zumeta et al., 2016), which in turn promoted personal well-being and social cohesion.

Considering the context-dependent nature of flow (Ghasemi et al., 2021), there is still a need to investigate EFL learners' flow in the transition to online education during the COVID-19 pandemic. Moreover, the existing body of research suggested the association between emotional intelligence components and flow in various contexts, including the foreign language setting (Dewaele et al., 2022), and studies on boredom in online classes during the COVID-19 pandemic pointed to the paramount importance of individuals' emotional experiences in second language context (Wang and Derakhshan, 2021; Kruk et al., 2022). Nevertheless, the possibility of enhancing EFL learners' experience of flow through EI intervention is unexplored and such relation in an online setting would provide useful insights for the language teachers. Thus, the main objective of this study was to investigate whether EFL learners' engagement with emotional intelligence tasks would be associated with a higher degree of flow. Accordingly, two research questions were posed in the current investigation:

1. Is it possible to nurture learners' emotional intelligence in an online EFL class?
2. What is the effect of using emotional tasks in improving EFL learners' perceived flow?

## Methodology

### Design of the study

**Figure 1:**
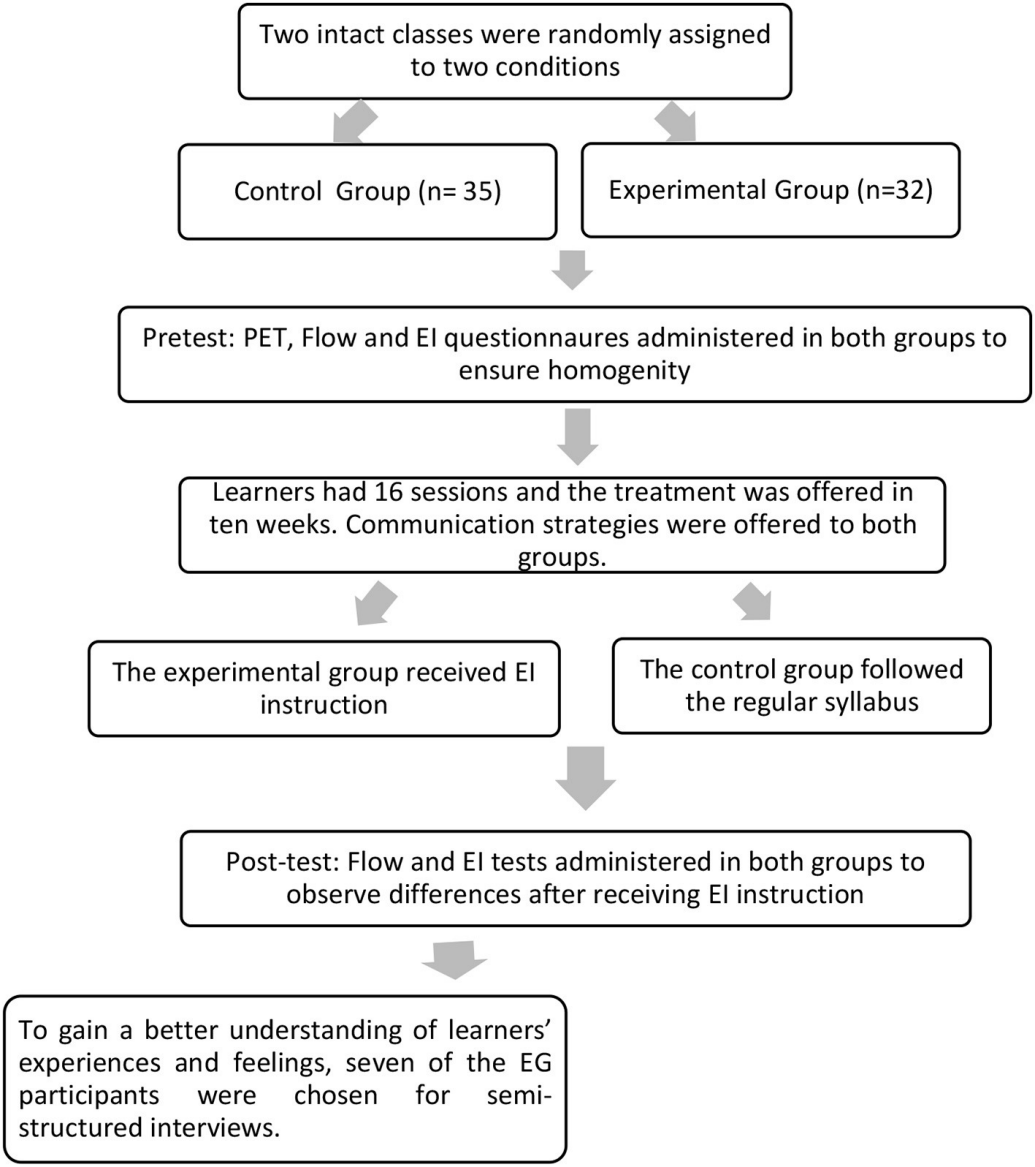
Design of the study.

This article is an attempt to enhance EFL learners' perceived flow through applying emotional intelligence activities. To do so, the teacher adopted various strategies to encourage social interaction and cooperative learning, motivate learners, and achieve deeper and more permanent learning. Therefore, the study adopted a mixed-method design to assess the effects of two teaching approaches on the learners' perceived flow in an EFL context. To this aim, we had two groups of learners going through different interventions. Figure 1 below shows the different stages of the study.

### Participants

A convenience sample of 67 Iranian EFL learners, studying English in a private language institute, participated in the study. They had been enrolled in two different upper-intermediate conversation classes and were randomly assigned to experimental (*n* = 32) and control (*n* = 35) groups. There were both male and female participants in both groups and they varied in age from 18 to 23 years old (M = 19.43, SD = 2.57). Participants mostly had studied English for about 6 to 8 years. Though the placement tests taken by the institute could provide enough assurance for their homogeneity regarding their general language ability prior to the study, the students were asked to take a Preliminary English Test (PET) and the results of the independent samples *t*-test confirmed their homogeneity. The participants were informed that participation was entirely voluntary, and were assured of the confidentiality of their responses.

### Instruments

To achieve the purposes of the current study, the following instruments and materials were utilized:

a. Preliminary English Test (PET): To check the homogeneity of the two groups, the listening and speaking parts of the Cambridge PET were used. The test shows the learners' capacity to achieve most goals and express themselves on a range of different topics. The listening section of the test consisted of 24 questions in four parts (namely pictures with multiple-choice questions, long recordings–e.g., interviews, academic lectures, etc.–, multiple-choice questions, complete notes and true/false). The speaking test included three parts (namely short questions and answers between the examinee and the examiner, looking at a visual aid and engaging in a discussion with the other candidate, and speaking about a picture for 1 min). In this study, the reliability of the test was estimated using Cronbach's Alpha and found to be 0.83.
b. *Trait Emotional Intelligence Questionnaire-Adolescent Short Form (TEIQue-ASF):* The short English version of the TEIQue (Petrides et al., 2006) including 30 items, which is a simplified version in terms of wording and syntactic complexity of the adult short form of the TEIQue, was used to measure learners' EI. All items are sampled from the 15 subscales of the adult trait EI sampling domain (two items per subscale). The test yields scores on four factors, namely well-being, self-control, emotionality, and sociability in addition to global trait EI. Some example items are “I can control my anger when I want to,” “I'm happy with my life,” and “I'm good at getting along with my classmates.” Higher scores on this scale imply higher levels of trait EI. Answers are collected on a 7-point Likert scale ranging from “Completely Disagree (number 1)” to “Completely Agree (number 7).” Cooper and Petrides (2010), applying item response theory, confirmed the validity of TEIQue-ASF. Following previous studies in the Iranian EFL context (e.g., Abdolrezapour and Tavakoli, 2012; Ghanbari and Abdolrezapour, 2021) which confirmed the reliability and validity of the scale, the instrument was validated in the particular context of the study. The reliability of the test was found to be high (Cronbach's α = 0.84).
c. *Egbert's (**2003**) inventory of learners' flow:* To measure learners' state of flow, Egbert's (2003) inventory of learners' flow was used (see Appendix A). The questionnaire required students to reflect on experiences during the preceding task and consisted of 14 items in four dimensions: four items for interest (e.g., “When doing this task, I was totally absorbed in what I was doing.”), three items for control (e.g., “This task allowed me to control what I was doing.”), three items for focus (e.g., “I would do this task even if it were not required.”), and four items for a balance of skills and challenges (e.g., “This task made me curious”). Learners were required to rate themselves on a seven-point Likert-type scale with response options ranging from 1 (strongly disagree) to 7 (strongly agree). Four of the questions were reversely scored. The higher score represents a higher level of flow experience and more concentration on learning tasks. In this study, the Cronbach's alpha values of the questionnaire and the four dimensions, i.e., interest, control, focus, and the balance of skills and challenge, were 0.85, 0.78, 0.84, 0.89, and 0.79, respectively. This instrument was supplemented with questions collecting demographic information including the participants' age, gender and self-report level of language proficiency.

#### Tasks

As the experience of flow differs in different skills, tasks, and activities (Ghasemi et al., 2021), to ensure higher comparability across the responses provided by learners, they were required to answer the items based on their experience of the language tasks and activities during which they had interactions with their friends. Accordingly, an oral narrative task was used, in which subjects were required to narrate a story based on a sequenced set of picture prompts. The tasks were previously piloted with a group of EFL learners of the same level and the expert colleagues confirmed their suitability for these participants based on Egbert (2003) criteria of the conditions facilitating the occurrence of flow, i.e., the perception of appropriate challenges, clear goals, an interesting task, sufficient time, immediate feedback, a sense of control and a chance to focus.

#### Semi-structured interviews

Following Egbert (2003, p. 508), who posited that “there is no objective way to measure flow precisely,” in addition to the flow questionnaire, to gain a more in-depth understanding of learners' experiences and feelings, an interview guide was developed addressing key issues related to the experience of flow. A significant advantage of this data collection approach is its suitability for revealing situation-specific factors or cultural characteristics of flow (Swann et al., 2012). Thus, seven EFL learners were then required to answer a number of questions in a semi-structured interview (see Appendix B). The incentive behind having semi-structured form of interview was to have a number of open-ended questions with a general guide with. In this way, we let the participants direct the interview and help address issues that were not previously considered by the authors. The interviews were audio-recorded with the consent of the participant and the recordings were transcribed and analyzed.

#### The EI intervention

As mentioned before, connecting learning tasks and activities to learners' personal lives would lead to deeper learning. Keeping this is mind and following Egbert (2003) who claimed that “teachers can theoretically facilitate the flow experience for students by developing tasks that might lead to flow” (p. 513), a 10-week intervention was developed for the experimental group which was integrated into the regular syllabus. So that the subjects received the regular English instruction in an hour and then 1-hour EII, whereas the control group received their regular 2-hour English instruction. The EII involved storytelling, reflective activities, group discussions, and experience-sharing. Emotional tasks and activities adopted in the study comprised the following components and were based on the five characteristics of EI proposed by Goleman (1995):

1. Helping learners know and manage their feelings: this characteristic of emotional intelligence gains more importance in online education where many learners find it difficult to cope with the many feelings they experience. Thus, learners were taught to identify their emotions and they were instructed to feel them rather than avoid them. So, the teacher encouraged learners to talk about the emotions they had during the hard times in the online education and their friends were encouraged to listen and give directions to them.
2. Guiding learners on strategies to know others and handle relationships: as most relationships are supposed to be limited in online education, there is an urgent need to work on strategies to maintain healthy relationships with peers and the teacher. Thus, to cultivate friendship among the individuals, they were guided to maintain a sense of self. Then, they were asked to discuss strategies they adopted to both stay connected to old relationships and form new ones in online classes. This strategy was hypothesized to help learners stay in tune with the teacher and the peers' conversations.
3. Teach learners monitor their progress: to enhance the learners' self-motivation, they were required to do some goal-setting tasks. They were also guided to revise and adjust the goals if they were not able to meet them.

#### Data collection procedure

First, the participants were pretested on language proficiency level, EI and flow states to ensure the sample homogeneity in terms of the variables under investigation. Next, those in the experimental group went through the EI intervention and those in the control group received the regular class activities with no special focus on emotional concepts and notions. For each session, the teacher had alternate exercises for both groups so that if the primary tasks were not interesting, a different approach or different tasks could be adopted. In addition, to gain a more in-depth understanding of the participants' classroom behavior and of their classroom learning experiences, seven students from the experimental group were chosen to participate in semi-structured interviews. The interviews were in Persian and were conducted by one of the researchers (a Persian native speaker) and lasted around 30–40 min each. Finally, post-tests (i.e., EI and flow measures) were administered to both groups.

#### Data analysis

Learners' EI and perceived flow were assessed using the quantitative methods and the SPSS software. So that first descriptive statistics were used and the obtained scores were checked in terms of the normality of distribution using measures such as Kurtosis and Skewness. Then, mixed between-within group ANOVAs were performed on the scores obtained from the pretests and posttests to examine the learners' improvement over time. Then, the learners' interviews were qualitatively analyzed to find the common themes within the transcripts. We adopted a three-step grounded-theory analysis (GTA) (Corbin and Strauss, 2008) of the interview responses, including open coding, axial coding, and selective coding. In the open coding stage, the themes expressed by the participants were coded by each researcher and data were organized based on the phrases and ideas that were stated and re-stated in the interviews. In this stage, if a component was discovered which was not in previous studies, the authors had to discuss it and reach an agreement to whether include it as a main category or a subcategory. Then, in the axial coding stage, relationships among the codes were identified and the links between categories and sub-categories were checked. Finally, in the selective coding stage, the transcripts and the selected codes were checked several times to select the codes underlying the students' perceived flow. To ensure the reliability of the obtained results, both intra-coder (by each researcher) and inter-coder (by both researchers) reliability checks were done. Moreover, to enhance the accuracy of the data, member checking or participant validation was performed, where participants were asked to check the transcripts of their interviews and correct any misinterpretations of their responses.

## Results and discussion

First, to check initial homogeneity of the groups, independent samples *t*-tests were run. The results of descriptive statistics and *t*-tests on EI and flow pretests are provided in Table 1. As shown, the results confirmed the comparability of the two groups prior to the intervention.

**Table 1:** Descriptive and inferential statistics on pretests.

**Variables**	**Group**	***N***	**Mean**	**SD**	**Sig**	**t**	**Mean difference**
EI	CG	35	113.69	7.16	0.798	−0.64	−1.18
	EG	32	112.50	7.79			
Flow	CG	35	43.28	5.83	0.216	−0.83	−1.28
	EG	32	42.00	6.79			

Level of significance is considered as p < 0.05.

### The impact of the intervention on learners' EI

To find the effect of the intervention on learners' EI, a mixed between-within group ANOVA was conducted on the pre- and posttest scores of the two groups, with the type of condition or treatment (i.e., experimental or control group) as the between-subjects factor and time (pretest and posttest scores) as the within-subjects factor. Table 2 displays the EI posttest descriptive statistics for each group, which point to the better performance of the experimental group on the posttest (Mean difference 24.72). The results of a mixed between-within group ANOVA showed main effects for time, *F*_(1, 65)_ = 262.177, *p* < 0.05, the interaction between the time and treatment condition, *F*_(1, 65)_ = 246.708, *p* < 0.05 (Table 3), and also the treatment condition, *F*_(1, 65)_ = 64.796, *p* < 0.05 (Table 4). These findings provide evidence that the learners improved over time as a result of the treatment condition and also that the two treatment conditions engendered differential effects on the learners' improvement in EI.

**Table 2:** Descriptive statistics of EI posttest scores.

**Variable**	**Group**	***N***	**Mean**	**SD**	**Std. Error Mean**	**Mean difference**
EI	CG	35	114.89	5.36	1.16	24.72
	EG	32	138.81	6.87	0.94	

**Table 3:** Tests of within-subjects effects for EI scores.

**Source**	**Pillai's trace**	**Type III sum of squares**	**df**	**Mean square**	**F**	**Sig**.
Time Time*Group	0.801	5964.064	1	5964.064	262.177	0.00
	0.891	5612.183	1	5612.183	246.708	0.00

Level of significance is considered as p <0.05.

**Table 4:** Tests of between-subjects effects.

**Source**	**Type III sum of squares**	**df**	**Mean square**	**F**	**Sig**.
Group	4631.969	1	4631.969	64.796	0.000

Level of significance is considered as p <0.05.

Thus, the mixed between-within group ANOVA confirmed the effectiveness of exposing language learners to emotional intelligence intervention on their emotional states. In other words, while a significant difference was observed between the pre-test and posttest EI scores of the experimental group, the EI scores of the control group did not increase significantly from pre-test to post-test conditions.

### The impact of the intervention on learners' perceived flow

As mentioned before, this article was an attempt to enhance EFL learners' perceived flow. So, to find a plausible answer to the second research question which aimed to investigate the impact of the EG activities on learners' perceived flow and to study the potential differences between the two groups of participants as well as the changes in the individual members of each group over time, a mixed between-within group ANOVA was run. Once again, the type of treatment was taken as the between-subjects factor and time was considered as the within-subjects factor. Learners' levels of perceived flow in the posttest are brought in Table 5, which, again, shows the better performance of the experimental group. The results of a mixed between-within group ANOVA showed main effects for time, *F*_(1, 65)_ = 22.682, *p* < 0.001 and the interaction between the time and treatment condition, *F*_(1, 65)_ = 22.355, *p* < 0.001 (Table 6); as well as the treatment condition, *F*_(1, 65)_ = 4.559, *p* < 0.001 (Table 7).

**Table 5:** Descriptive statistics of flow posttest scores.

**Variable**	**Group**	***N***	**Mean**	**SD**	**Std. error mean**	**Mean difference**
Flow	CG	35	43.31	5.54	0.93	6.56
	EG	32	49.87	6.17	1.09	

**Table 6:** Tests of within-subjects effects for flow scores.

**Source**	**Pillai's trace**	**Type III sum of squares**	**Df**	**Mean square**	**F**	**Sig**.
Time Time*Group	0.259	522.108	1	522.108	22.682	0.000
	0.256	514.585	1	514.585	22.355	0.000

Level of significance is considered as p <0.05.

**Table 7:** Tests of between-subjects effects for flow scores.

**Source**	**Type III sum of squares**	**Df**	**Mean square**	**F**	**Sig**.
Group	232.572	1	232.572	4.559	0.037

Level of significance is considered as p <0.05.

According to the results of this part of the study, the students differed in their flow condition based on the particular group they belonged to. While a significant improvement was found in the flow scores of the students in experimental group, the flow condition of the students in the control group did not differ significantly from pre-test to post-test occasions.

### Semi-structured interviews

This part of the article provides the information obtained from interviewing seven EFL learners participating in the experimental intervention. As mentioned in the previous section, the interviews were transcribed and coded applying the GTA. From the in-depth interview data, which aimed to provide insight into the signs of flow experience, several coding themes were identified, including enjoyment, engagement with the tasks, reduced self-consciousness, and intense concentration. Table 8 shows the frequency of the final categories observed in the interview transcript of each single student. It should be pointed that if a student pointed to a particular category several times, it was only counted once.

**Table 8:** Coding categories of the signs of flow experience in interview data with representative extracts.

**Category**	**Representative extracts**	**Frequency**	**Percentage**
Enjoyment	This enjoyable experience was one of a kind. The task was interesting and manageable.	7	100%
Engagement with the tasks	I was willing to do the activity several times. I knew what I was expected to learn.	6	86%
Loss of self-consciousness	I wasn't worried about making mistakes. I was lost in the activity. I felt like time flew.	5	71%
Intense concentration	I was totally absorbed in the tasks.	6	86%

Here, the analysis of the interviews along with some excerpts from the participants that showed the existence of flow state after completing language activities would be provided. As noted above, the adopted tasks and activities were mainly based on the Goleman (1995) model of emotional intelligence; however, based on the results of semi-structured interviews, these tasks led to the main dimensions of flow including enjoyment, engagement with the tasks, concentration, reduced self-consciousness and absence of time alertness. However, as stated above, some components were taken as the subcategory, rather than a new category to conform with the literature. Thus, relationships among the codes were identified and absence of time alertness was considered as the sub-category of reduced self-consciousness. Moreover, participants (either implicitly or explicitly) referred to the balance of skill and challenge, clear and focused goals, and the feedback provided by the teacher as the main antecedents of their perceived flow.

In general, the EI intervention was found to engage the learners' interest as some commented that they liked the goals of the tasks and the feedback received made them sure that they were performing the tasks properly as one commented:

I really like to replicate the tasks as the teacher was telling us exactly what we had to do and she said that we should have a goal in listening and speaking tasks. The ongoing feedback that she was giving us made the course much more interesting. We knew whether we are moving toward the goal or we have to revise our performance.

This part was in line with what Egbert (2003) posited, i.e., tasks presented in language classroom should have clear goals to induce learners' flow. Participants also proposed that they were more interested in doing the tasks and experienced loss of self-consciousness which is reflected in the excerpt below stated by another learner:

The activities were connected to our lives so we were more engaged. As for me, I had lower stress and my mind was relaxed and I could perform with much more ease.

Learners also enjoyed the collaborative tasks and the experience of talking about emotions. They liked the degree of challenge they had, which was once again in line with what Egbert (2003) mentioned, i.e., to attain learners' flow, there should be a balance of skills and challenge. An example of such experience can be observed in the following comment provided by one learner:

The tasks were neither hard nor easy; sometimes we had some collaborative tasks for which we had to discuss the answer in the WhatsApp group and share the results with other classmates. In such cases and the times when we were listening to the stories of our friends, we couldn't perceive the passage of time.

Furthermore, the tasks and activities offered to language learners resulted in their intense concentration; an instance is shown in the following comment:

For some tasks, we were given a time to talk together and share our feelings about the things happened to a friend. The thing that I liked about such tasks was that the teacher didn't interrupt us and we could focus on the activity, sometimes we even forgot that we were taking part in a formal class. The tasks were wonderful.

## Discussion

As noted above, this study was an attempt to, first, explore the impact of the application of an EI intervention program on the EFL learners' emotional intelligence level, and second, to examine the extent to which this intervention would impact the learners' perceived flow. The results pertaining to the first research question, which concerned the possibility of nurturing learners' emotional intelligence over the 10-week program, showed that exposing EFL learners to the intervention positively affected their emotional intelligence level. The results of this part of study confirmed the literature regarding the possibility of nurturing emotional intelligence in various educational settings (Abdolrezapour, 2017a; Dewaele et al., 2018; Ghanbari and Abdolrezapour, 2021) and were in line with the studies which pointed to the predictive contribution of learners' emotional intelligence in explaining variance in their readiness for online education (Buzdar et al., 2016; Abdolrezapour, 2017b; Fraschini and Tao, 2021). Thus, teaching intrapersonal emotional factors such as managing one's emotions, knowing one's emotions and motivating oneself in addition to the interpersonal emotional characteristics such as understanding others' feelings and strategies to handle ones' emotions would be beneficial to language learners in online educational settings.

The second research question addressed the effect of the EI intervention on nurturing EFL learners' flow. The findings provided evidence that the learners improved over time as a result of the treatment condition and also that the two treatment conditions engendered differential effects on the learners' improvement in flow, which confirmed Pearce (2005) claim of the possibility of making e-learning activities motivating and engaging. In line with the L2 literature that pointed to the task type and task engagement as the common boredom-inducing factors in online education (Derakhshan et al., 2021b; Zawodniak et al., 2021; Kruk et al., 2022) who suggested adopting educational programs with improved interpersonal relationships and more teacher-student interactions, incorporating the EI intervention with the different topics and activities made language learners more willing to participate. Moreover, according to Csikszentmihalyi (1998), when individuals participate in an activity for its own sake, i.e., it is so satisfying that they are inclined to repeat it at higher levels of challenge, their perceived flow would increase. So, it seems that learners' exposure to various emotional tasks and activities made them more interested and eager to participate in activities and this accordingly increased their flow.

Reasons for the difference in learners' perceived flow over a 10-week period were provided in the semi-structured interview sessions with the open-ended questions, which were intended to look into the experimental group's perceptions of the EI intervention and signs of enhanced flow. Our qualitative data showed that participants were more willing to participate in educational tasks and share their feelings and thoughts with their classmates. The intervention applied in this study led to enjoyment, engagement with the tasks, reduced self-consciousness, and intense concentration. So, considering the results obtained in this part of the study and the literature (e.g., Rodriguez-Ardura and Meseguer-Artola, 2017; Dewaele and MacIntyre, 2019; Li et al., 2021; Liu and Song, 2021; Wang and Huang, 2022; Zhao and Khan, 2022) which confirmed the efficiency of flow in the foreign language context, it can be a good strategy to expose language learners to teachable techniques for managing emotions, knowing one's emotions, handling relationships, and motivating oneself.

## Conclusion

During traumatic circumstances, such as the COVID-19 pandemic, many individuals might not be in a desired emotional state to focus on educational tasks and activities. In such situations, online educational programs, capable of driving students' interest and effort, lead to higher learning outcomes. Learners who experience flow are more eager to participate actively in learning tasks and have one predetermined learning objective; they set a goal for themselves and feel more pleasure and are much more satisfied. In such a state, they do not consider other thoughts and distractions. Thus, to ensure learning success, it is necessary to develop learning tasks and activities that yield favorable opportunities to all language learners through providing an optimal level of challenge, control, and interest.

Following Goleman (1995), who posited that flow state is resulted from the highest level of emotional intelligence, students' eagerness and performance in online learning can be ameliorated through regulating their emotions and raising their emotional intelligence. And as learners with higher levels of EI have higher self-awareness, self-management, and are more capable of handling social relationships, they can ultimately perform better in online educational courses that inherently abound with internal distractions (including learners' feelings and interference from homes such as younger siblings and parents), and external ones (such as other students and technical complexities such as difficulties experienced in connecting to the net).

In line with previous studies (Abdolrezapour, 2017a,b; Ghanbari and Abdolrezapour, 2021), this study introduced some emotional activities to provide opportunities for language learners to work on emotional intelligence, and such activities can be adopted in various educational contexts. Generally, focusing on learners' emotional states and designing tasks to discuss such issues in the class will allow learners to see the lighter and more humorous side of things, and to regain some qualities as persistence, motivation, willingness, and cheerfulness that characterize successful learners. In addition, it would increase their inner peace and strength, and in doing so, it helps them be able to control and reduce the stresses that accompany some learning contexts, especially virtual ones. When working on learners' emotional intelligence, they can develop their vision and lower their stress, fear, and disappointment. They can have a much more positive attitude and experience lower levels of negative feelings. Thus, considering the teacher's crucial role in setting the conditions for ensuring positive flow (Dewaele and MacIntyre, 2019), it is suggested that teachers apply various educational tools and different tasks to make learners more engaged and interested and increase their motivation.

As with all other studies, this study has a number of limitations that need to be acknowledged. First, we had a small sample for semi-structured interviews and only from the experimental group, which might delimit the generalizability of the study. Future attempts can strengthen the generalizability by conducting similar research with more in-depth investigation of learners' views from both the control and experimental groups. Also, the generalizability of the obtained results is subject to certain limitations as the study used a convenience sample. Finally, to expand the use of emotional intelligence intervention in various educational settings and especially in online settings, the provision of a more comprehensive educational program can also be a productive venue for future research.

## Data availability statement

The data that support the findings of this study are available from the corresponding author upon reasonable request.

## Ethics statement

The study was reviewed and approved by Research Ethics Committee of the Salman Farsi University of Kazerun. All procedures performed in the study were in accordance with the ethical guidelines of the 1964 Helsinki declaration. In addition, at the onset of the study, our participants were informed that the data of the study will be used only for research purposes and that all data will be used anonymously. The participants provided their written informed consent to participate in this study.

## Author contributions

PA prepared the approach and instruments, she was also the major contributor in writing the manuscript. NG was the instructor of the two classes, collected the required data, and interpreted all data. Both authors read, revised, and approved the final manuscript.

## Conflict of interest

The authors declare that the research was conducted in the absence of any commercial or financial relationships that could be construed as a potential conflict of interest.

## Publisher's note

All claims expressed in this article are solely those of the authors and do not necessarily represent those of their affiliated organizations, or those of the publisher, the editors and the reviewers. Any product that may be evaluated in this article, or claim that may be made by its manufacturer, is not guaranteed or endorsed by the publisher.

## Appendix

### Appendix A

Biographical information:

Gender: female () male ()

Age: ___ years

How long have you been learning English? For ___ years.

Language proficiency level: Lower intermediate (), intermediate (), advanced ()

Egbert's (2003) Flow Questionnaire

*Please think about the task you have just done. How did you feel during the task?*

1. This task excited my curiosity.

2. This task was interesting in itself.

3. I felt that I had no control over what was happening during this task.

4. When doing this task, I was aware of distractions.

5. This task made me curious.

6. This task was fun for me.

7. I would do this task again.

8. This task allowed me to control what I was doing.

9. When doing this task, I was totally absorbed in what I was doing.

10. This task bored me.

11. During this task, I could make decisions about what to study, how to study it, and/or with whom to study.

12. When doing this task, I thought about other things.

13. This task aroused my imagination.

14. I would do this task even if it were not required.

### Appendix B

#### Interview guide

**Please answer the following questions**

In some situations, you find yourself completely engaged in an activity to the extent that your worries, sense of time, and self-consciousness seem to disappear and you lose track of time.

As for the purpose of this study, we seek the moments in which you lose your sense of time, you're completely enraptured, you're completely caught up in what you are doing in online EFL courses. So, please answer the following questions?

- How did you feel about this class?

- Have you ever had an experience like this in your online classes? Please describe it.

- In as much detail as possible, can you describe what these experiences look like?

- If you were given the opportunity to do this task again, would you?

- Which flow-inducing factors make a difference between online and traditional classes?

- What are the most distinguishing characteristics, or clearest indicators of being in flow?

- What prevents flow? Are there times when the flow is more or less likely to occur?
